# DMFL_Net: A Federated Learning-Based Framework for the Classification of COVID-19 from Multiple Chest Diseases Using X-rays

**DOI:** 10.3390/s23020743

**Published:** 2023-01-09

**Authors:** Hassaan Malik, Ahmad Naeem, Rizwan Ali Naqvi, Woong-Kee Loh

**Affiliations:** 1 Department of Computer Science, University of Management and Technology, Lahore 54000, Pakistan; 2 Department of Unmanned Vehicle Engineering, Sejong University, Seoul 05006, Republic of Korea; 3 School of Computing, Gachon University, Seongnam 13120, Republic of Korea

**Keywords:** federated learning, COVID-19, TB, lung cancer, data privacy, machine learning

## Abstract

Coronavirus Disease 2019 (COVID-19) is still a threat to global health and safety, and it is anticipated that deep learning (DL) will be the most effective way of detecting COVID-19 and other chest diseases such as lung cancer (LC), tuberculosis (TB), pneumothorax (PneuTh), and pneumonia (Pneu). However, data sharing across hospitals is hampered by patients’ right to privacy, leading to unexpected results from deep neural network (DNN) models. Federated learning (FL) is a game-changing concept since it allows clients to train models together without sharing their source data with anybody else. Few studies, however, focus on improving the model’s accuracy and stability, whereas most existing FL-based COVID-19 detection techniques aim to maximize secondary objectives such as latency, energy usage, and privacy. In this work, we design a novel model named decision-making-based federated learning network (DMFL_Net) for medical diagnostic image analysis to distinguish COVID-19 from four distinct chest disorders including LC, TB, PneuTh, and Pneu. The DMFL_Net model that has been suggested gathers data from a variety of hospitals, constructs the model using the DenseNet-169, and produces accurate predictions from information that is kept secure and only released to authorized individuals. Extensive experiments were carried out with chest X-rays (CXR), and the performance of the proposed model was compared with two transfer learning (TL) models, i.e., VGG-19 and VGG-16 in terms of accuracy (ACC), precision (PRE), recall (REC), specificity (SPF), and F1-measure. Additionally, the DMFL_Net model is also compared with the default FL configurations. The proposed DMFL_Net + DenseNet-169 model achieves an accuracy of 98.45% and outperforms other approaches in classifying COVID-19 from four chest diseases and successfully protects the privacy of the data among diverse clients.

## 1. Introduction

Researchers have identified the coronavirus (COVID-19) pandemic as one of the most devastating health crises now impacting millions of people worldwide [1,2,3]. When an infected person coughs, sneezes, or talks, respiratory particles are released into the air that can easily spread the COVID-19 virus. An infected person may exhibit adverse symptoms such as a dry cough, fever, gastrointestinal symptoms, myalgia, and anosmia, which pose a significant threat to global health security [4]. Timely COVID-19 detection is critical for limiting the potential for widespread infections. As the foundation of artificial intelligence (AI) [5], machine learning (ML) [6] technology has seen extensive use by researchers in the medical treatment sector (e.g., health monitoring), and it is also expected to be an effective method for the efficient detection of COVID-19 and chest diseases, in particular, deep learning (DL) technologies on medical diagnostic images such as chest X-ray (CXR) images [7,8]. On the other hand, medical institutions, the owners of CXR images, are known to prefer training models on their data. This is because medical data are subject to stringent privacy requirements [9,10,11], which makes it difficult for medical institutions with fewer samples to train a model with the expected performance for detecting various chest diseases. General deep neural network (DNN) models are well known for general image classification; however, they frequently perform poorly with imbalanced datasets, as discussed in [6], and models trained on restricted samples typically have low generalization capabilities due to a lack of sample diversity [10,11,12].

This problem may be effectively mitigated by using transfer learning (TL) and federated learning (FL) [7], in which medical institutions at all network locations utilize local data for training without centralizing all of the data [10]. In 2016, Google initially presented the idea of FL as a fresh approach to ML. FL enables the building of global models without compromising the confidentiality of the underlying data [1,2]. During each training cycle, a subset of clients is selected to train a model with the data that are specific to their location. The modifications that were made to the local models are subsequently transferred to a centralized server to be aggregated; however, the raw data from the local servers are not sent. FL provides the opportunity to link together many medical institutions and construct a model for the positive case identification of COVID-19 from multiple chest diseases, and maintaining the confidentiality of the data. Recent studies [4,5,6,7,8,9] have shown that FL may be used to accurately diagnose COVID-19 by using X-ray images. However, the aforementioned studies used the default setup of FL, which results in poor performance when client data heterogeneity is present and requires significant communication costs for the transmission of model updates (for example, enormous weight matrices).

In light of the significant communication costs associated with parameter transmission, a decision-making-based federated learning network (DMFL_Net) framework has been proposed. Within this framework, clients are dynamically selected to participate in each global training by configuring the request protocol between the server and the clients. As a result, communication efficiency during training has been significantly improved [13]. A blockchain-based solution was integrated with its decentralized feature [14] to decentralize the aggregation process to improve the privacy of data analysis in the identification of federated COVID-19 and four chest illnesses. This was done to enhance the confidentiality of the data. To provide performance-observable services for the COVID-19 detection system that is based on FL, a real-time method that is capable of assessing the contributions of FL framework participants in real-time is expected to be developed. However, the existing FL-based COVID-19 and chest disease detection algorithms have the propensity to employ the most prevalent techniques FedAvg [15], and maximize the number of objectives, including latency, energy use, and privacy. There have only been a few studies [5,6,7,8,9,10,11,12,13,14,15,16] that have looked at the real-world distribution characteristics of COVID-19 instances and built a one-of-a-kind central aggregation method. This strategy achieves a successful adjustment to the complex sample environment of COVID-19 and at the same time focuses on enhancing the accuracy and stability of the global model. It is vital, in the area of medical detection, to establish a higher number of possible instances and assure the consistency of the model across all of the exams.

### 1.1. Motivation

The most recent study conducted by the World Health Organization (WHO) indicates that COVID-19 is an infectious illness that mostly affects the lungs, giving them a honeycomb-like appearance as a result. Even after making a full recovery from COVID-19, some people are nevertheless left with persistent lung impairment. The primary purpose of our research was to characterize the lung patterns brought on by COVID-19 to ensure that highly trained radiologists do not overlook infection. Second, the sharing of information to train a more effective DNN model and take into account the privacy concerns of data sources. It is possible to develop a DNN model for automated COVID-19 identification from four other chest diseases as part of the data-sharing process.

The fact that private information cannot be shared is the first roadblock, which arises from the absence of privacy protections. The second challenge is to train the global model via the use of an FL network. It is difficult to obtain enough training data and improve the prediction model, both of which have an effect on the diagnosis ratio. Last but not least, recognizing the COVID-19 lung screening pattern is a challenging problem. This problem inspired the creation of a one-of-a-kind model for the DMFL_Net that can diagnose instances of COVID-19 and share the data while simultaneously improving hospital communication, privacy, and security.

### 1.2. Contribution

To improve both the efficacy of communication and the performance of the model, we provide a novel model DMFL_Net for the classification of COVID-19 from four different chest diseases, i.e., lung cancer (LC), tuberculosis (TB), pneumothorax (PneuTh), and pneumonia (Pneu) using CXR images. To the best of our knowledge, this is the only study that uses FL on CXR for the classification of COVID-19 from four different chest diseases. The following are the key contributions of the present study:

1. To distinguish COVID-19-positive cases from other chest diseases, we build a DMFL_Net architecture for medical diagnostic image processing. The proposed design provides a rational depiction of the interactions between the components and serves as a guideline for the development of the FL system.
2. We introduce a DMFL_Net that schedules model combinations under the amount of time each client spends in training and selects clients to participate based on the performance of their local models. Every client evaluates the locally trained model, and updates are only sent to the server if they improve the model’s overall performance. Based on the normal amount of time spent training during the prior cycle, the central server computes the amount of time spent waiting for each client to submit model updates. This calculation is done based on the total amount of time spent training.
3. We provide an overview of a category of medical diagnostic image data sets for COVID-19 identification from four chest disorders. These image data sets may be exploited by the community of ML researchers to research image analysis.
4. The evaluation demonstrates that the proposed method is an advance over the current state-of-the-art for FL in terms of accuracy (ACC), recall (REC), precision (PRE), f1-measure, specificity (SPF), and efficiency of communication.

This research paper proposed a DMFL_Net framework with the goals of improving the identification of multiple-source CXR images, facilitating the safe exchange of data among institutions, and doing all of this while maintaining patient anonymity. In addition, DNN models such as DenseNet-169, VGG-16, and VGG-19 are used for efficient feature extraction and classification to differentiate COVID-19 from four distinct chest disorders that are present in the various CXR image datasets that are available to the public.

### 1.3. Structure of Paper

This work structure is divided into further sections. Section 2 discusses the modern literature on COVID-19 detection using FL methods. Section 3 presents the methodology of the study. Section 4 consists of results and discussions. This study is concluded in Section 5.

## 2. Literature Review

The study and development of DL classification algorithms [15,16,17] for COVID-19 using X-rays have received a significant amount of attention and effort over the past few years. But, in 2016, Google introduced the concept of FL [1], which primarily concentrates on learning across several devices. In the beginning, Google made predictions for search recommendations, next words, and emojis using FL [13,14,15]. After then, the purview of FL was broadened to include cross-silo learning, for example, for several different companies or data centers [16,17,18,19,20]. A segmentation model was built by Sheller et al. [21] with the assistance of data on brain tumors obtained from a variety of different medical institutes. Edge computing is another area where FL could be used, for instance in the task scheduling method for the Internet of Vehicles [13,14,15,16]. During training, FL systems required a large number of rounds of communication to achieve model convergence, even if the communication process could be made more efficient by providing just model updates rather than raw input. This is the case even though sending only model updates rather than raw data can improve the efficiency of communication. A large number of scholars are investigating a variety of approaches to cut down on the duration of communication cycles [22,23]. Most of the studies [24,25,26,27,28] used a technique of aggregation, such as aggregation scheduling [20], asynchronous aggregation [21], and selective aggregation [14]. Moreover, compression techniques are employed to lower the model’s communication expenses between clients and servers communicate parameters and gradients to the central server [29]. In addition, ways of communication that are offered to enhance the effectiveness of communication include the over-the-air calculating approach [26] and the multichannel random access communication mechanism [29]. The effectiveness of communication was one of the primary motivations behind the development of both of these approaches.

ML models are trained locally, and FL can handle challenges related to statistical heterogeneity and system heterogeneity [30]. Despite this, there are still difficulties associated with making use of independent data that are non-identically distributed (non-IID). Numerous academics have conducted research on a variety of topics, including data clustering for training [31] and multitask learning [32]. In addition, the development of incentive systems to encourage consumer participation in ML developments has been the subject of research in several studies [33,34]. In more recent times, FL has been implemented in X-ray image processing to identify positive COVID-19 cases [35,36].

Shokri and Shmatikov [37] designed a decentralized architecture that gives users the ability to combine their gradients to increase the data’s level of security as they are working with them. As a consequence of this, there is a possibility that the information will be safeguarded in a manner that is more effective overall. On the other hand, even attackers who took a passive approach were able to profit from vulnerabilities in the strategies they used [38,39]. By utilizing the FL global model, Bonawitz et al. [40] were able to construct a framework for the secret aggregation of gradients. To prevent any changes to the gradients, Zhang et al. [41] devised threshold secret sharing and homomorphic encryption (HE). However, user identification is not possible when utilizing a shared model. Multiple inputs continue to be plagued by trust challenges, which puts the integrity of the data at risk and leads to insufficient training of the model. Xu et al. [42] were able to correctly classify X-rays as either normal or abnormal by employing a hierarchical convolutional neural network (CNN). Gupta et al. [43] proposed using a stacked CNN to make the diagnosis.

Recent research [44,45] has brought to light the significance of FL as an AI architecture that can be deployed and used at the edge of the network. When deployed in a setting that is heterogeneous and contains data that are non-IID, the centralized character of classic AI algorithms is in direct contrast to the decentralized nature of FL. Several distinct approaches have been developed to tackle the aggregation of non-IID data. These approaches make use of a variety of aggregation algorithms, such as FedMA [46], feature fusion [47], and grouping of related client models [39,48,49]. In the clustering process, similarity among client models is exploited [27], and encouraging excellent communication is one of the ways that data generalization is increased [22].

Feki et al. [17] built a collaborative FL architecture that allows medical institutions to filter COVID-19 from CXR images using DL without releasing patient data. They investigate a variety of crucial elements and characteristics of FL situations, such as the naturally occurring non-independent, non-IID and unbalanced data distributions. Their findings motivate medical institutions to rapidly establish a strong model for COVID-19 screening [50] by using collaborative procedures and the abundance of accessible private data. Chowdhary et al. [51] provided a technique that allows users to rapidly detect COVID-19 [52] by just uploading a single CXR photo in a matter of seconds. The StreamLit was used throughout the development of the front end, and the Flower framework was used for the development of the back end. The proposed methodology has proven successful after going through training for three iterations of federated communication, resulting in significant results. Ahmed et al. [53] suggested a DL classification model for COVID-19 based on CXR. After running 5-fold cross-validation on a multi-class dataset comprising of COVID-19, Viral Pneu, and normal CXR, their suggested DL model achieved an average classification accuracy of 90.64% and F1-Score of 89.8%. Based on computed tomography (CT) scans, Khan et al. [54] proposed an optimum DL approach as a means of distinguishing COVID-19-infected patients from healthy patients. To improve the overall quality of the images that were taken in their original form, the contrast was increased. An accuracy rate of 94.76% was achieved on average by using the proposed pretrained DenseNet-201 classification model. Albahli et al. [55] designed an AI-driven deep and handcrafted features selection approach for the identification of COVID-19 and chest-related diseases. Their proposed model achieved an average accuracy of 90.22% in classifying chest diseases using CXR. Verma et al. [56] established a DL model for the identification of COVID-19 in influenza-A virus cases and healthy patients by making use of patient pulmonary CT scans. A total of 548 CT scans were performed, with 232 coming from the bodies of 12 patients infected with COVID-19, 186 coming from the bodies of 17 patients infected with the influenza A virus, and 130 coming from the bodies of 15 healthy people who were not affected. To a degree of 79.39% accuracy, the model that was proposed performed adequately. 

The aforementioned study, on the other hand, does not address the issues of FL, namely those of communication efficiency and model accuracy. As a result, the findings of our research provided a strategy based on DMFL_Net for improving the efficiency of communication and the performance of models. Table 1 presents the recent literature on FL for the diagnosis of COVID-19.

## 3. Materials and Methods

In this section, we provide a DMFL_Net approach for evaluating chest X-ray images to identify COVID-19 from four chest illnesses, namely LC, TB, PneuTh, and Pneu. We discuss the individual components of the architecture as well as the interactions that occur between those components. In addition, a DMFL_Net technique is provided for dynamically determining the clients who will be participating and scheduling the aggregate following the amount of time that will be spent training each client.

### 3.1. Dataset Description

**Figure 1:**
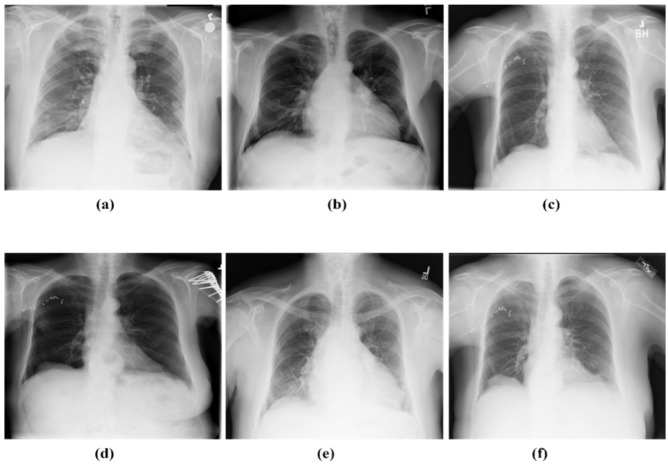
Sample images of CXR (**a**) TB, (**b**) LC, (**c**) COVID-19, (**d**) normal, (**e**) PneuTh, and (**f**) Pneu.

The authors of this study acquired a range of chest disorder datasets from several sources to train three pre-trained models, namely VGG-16, VGG-19, and DenseNet-169. Cohen et al. [57] created a GitHub repository, and it was through this that we were able to receive the CXR that were infected with COVID-19. CXR used in the creation of this database came from a wide variety of public and private institutions, both domestic and international, including hospitals and clinics. The average age of patients infected with COVID-19 was 55 years old; however, we do not currently have access to the complete set of metadata information. Through a search of the SIRM database [58], the TCIA database [59], radiopaedia.org [60], Mendeley [61], and the source on GitHub [62], we were able to collect a total of 2371 COVID-19-infected CXR. During the process of acquiring the CXR images collected for pneumonia, the RSNA [63] was utilized. This data set has a total of 5216 X-rays, of which 1349 were determined to be normal and the remaining 3867 revealed pneumonia. Utilization of the reference [64] allowed the acquisition of access to 5000 CXR of lung cancer. The CXR of healthy people was obtained from the Kaggle [65]. A total of 700 CXR images of TB were collected [66]. Figure 1 presents the sample images collected from the mentioned databases.

### 3.2. Data Augmentation and Segregating for Training and Testing

Table 2 shows that the CXR images of the four chest diseases and COVID-19 are imbalanced. Therefore, the synthetic minority oversampling technique (SMOTE) [28] technique is applied to increase the size of the dataset. The reason behind using SMOTE is to achieve a more equitable allocation of classes by iteratively and randomly multiplying situations involving minority class members. To train the three TL models (VGG-16, VGG-19, and DenseNet-169), we split the dataset into three sections; training, validation, and testing. As a result, only 10% of the CXR images were used for testing, and the remaining 80% and 10% were used for training and validation, respectively.

### 3.3. FL Architecture

**Figure 2:**
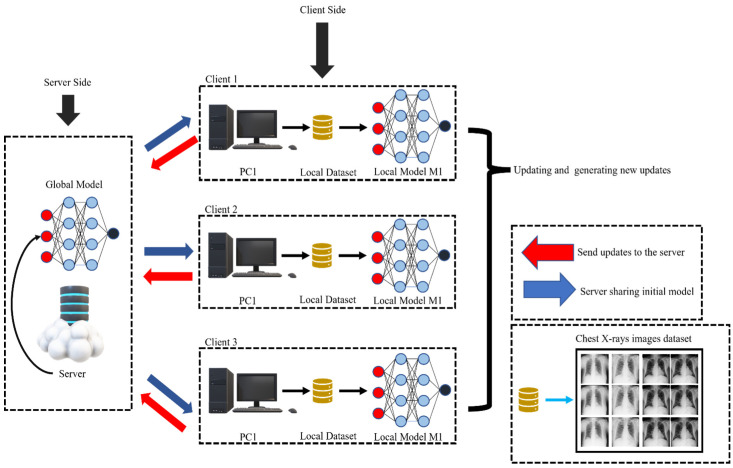
Framework for FL in CXR image processing for multiple chest diseases.

Figure 2 is an illustration of an architecture made up of two distinct types of components: (1) a central server, and (2) clients. The training of models that are stated in the learning task is the responsibility of the clients, who are responsible for utilizing local data and computational resources to train the models [67]. The centralized server, which is also responsible for managing the FL process, is the one that initiates the ML job. Using the client data collector, each client gathers CXR that have been scanned by imaging equipment, cleans the data using the client data preprocessor, and stores the information locally. This procedure is repeated for each client [68]. Once a model training task has been started, the starting waiting time for each client to return model updates can be modified by the job designer. This is possible even after the task has already begun (which includes the initial model code and several aggregations). Every client who participates in the activity first downloads the task, and then they train the model by making use of the model trainer [5]. Once the model trainer has completed the required number of epochs for this training round, the training time will be sent to the primary server [69]. The entire amount of training time that was collected from the participating clients is taken into account by the aggregation scheduler when determining how much time should be spent waiting. The performance of the most recent version of the local model is evaluated by the local model assessor and compared to the performance of the version that came before it for each client. If it is determined that the performance of the locally stored model that is currently accessible is superior, the client will submit a request to the primary server to upload the model. In that event, the client will request that the model update not be submitted for this particular round of testing. This aggregation cycle does not include any clients who fulfilled the necessary conditions, such as completing the required number of epochs within the allocated time [35]. The aggregate scheduler, which is situated on the central server, will send a notification to clients who have previously requested the model upload after the given period has gone. After the aggregation procedure has been finished, the global model assessor will evaluate the accuracy of the aggregated global model before sending it back to each client for an additional round of training.

### 3.4. Classification of COVID-19 Using DenseNet-169

As a result of a comparison of the experimental findings of COVID-19 with several CNN models in [70,71], DenseNet-169 was selected as the training model to be deployed in each client because it had the highest performance among all of the test models, i.e., VGG-16 and VGG-19 as discussed in this article. As shown in Figure 1, once all iterations on the global level have finished, the central server will send the final global parameters to each client for testing. In our experiment, the preprocessed CXR images serve as the input for the local DenseNet-169 model, which is fitted using global parameters. On the other hand, 299 × 299 pixel matrices serve as the input for the local DenseNet-169 model. Each sample’s model output is a three-dimensional vector representing the likelihood of each type, and optimal detection may be achieved by optimizing the value function.

### 3.5. Proposed DMFL_Net

**Figure 3:**
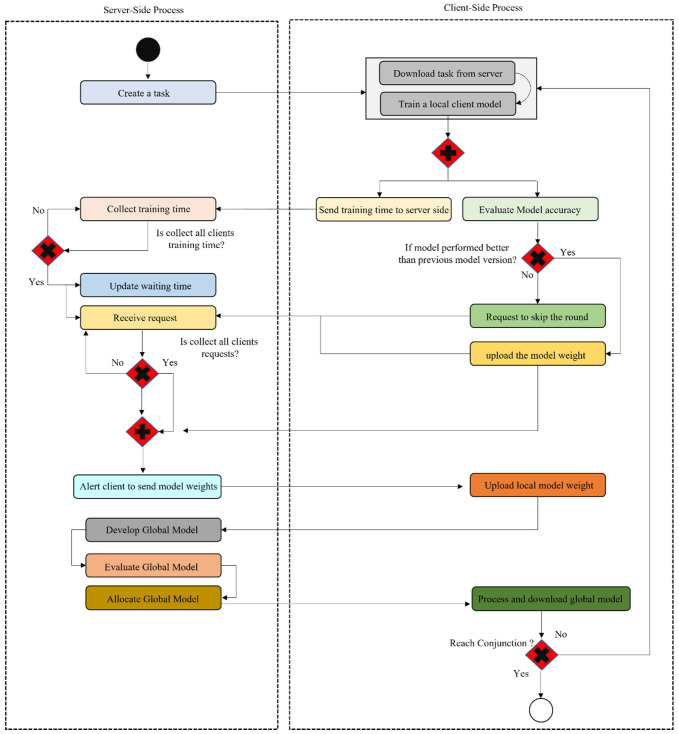
Process of proposed DMFL_Net.

The DMFL_Net model that has been suggested comprises two decision-making points: (1) client participation, and (2) client selection. These decision-making points are included to increase the efficiency of communication in FL. When determining whether or not to take part in the current round of aggregation, each client takes into account the performance of the newly trained model to determine which option best suits their needs. The model aggregator that is stored on the primary server selects which clients will participate based on the amount of time those clients spend waiting for their turn. The length of the first waiting time is determined by the proprietor of the platform. The waiting time for a client in the current round is computed by taking the client’s average training time from the round before. The central server will remove a client from participation in this round of aggregation if the client is unable to successfully upload the model update before the waiting period ends. The DMFL_Net approach that has been suggested is depicted in Figure 3. When a learning job is first created, it is done so by the central server. Before a client may create their local training environment, they must first retrieve the work from the centralized server and bring it back to their machine. A timer is started for each client at the beginning of the second round. The length of this timer is determined by the average amount of training time that was completed by the participants in the round before this one. If a client does not finish their training within the specified period, the centralized server will move through the process of aggregation for the current round without the client’s participation. The client will request that the aggregate for this round be skipped from the central server if it believes that the model it has trained for this round will do less well than the model it trained for the round before it. If not, then the client will alert the main server to do the model update.

When the proposed DMFL_Net starts training for the classification of COVID-19, the client’s training set is comprised of all of the datasets that were obtained by the CXR devices and is denoted by the D parameter. Following the client’s successful completion of the training set update, the training task will be carried out with DenseNet-169 with optimal settings that provide the least amount of training loss [72]. The global parameter that is provided by the central server is always loaded into local models as the very first parameter. Equation (1) represents the loss function of the client *C_LOSS_*:

(1) $$C_{LOSS}\left(W\right)=-\frac{1}{\left|D\right|}\underset{X\;\in \;D}{\sum }\overset{a}{\underset{a=1}{\sum }}B_{\left(X,\;a\right)}\times log\left(Q_{\left(X,a\right)}\right)$$

where the parameter a represents the number of data categories, W is used as the model parameter, *D* is the training set of the client *C_LOSS_*, *B_(X,a)_*, and *Q_(X,a)_* are applied for real and predicted labels, respectively. The gradient descent technique is applied to update *W* at each local iteration. Equation (2) is used for calculating the gradient descent method at iteration (*i*, *j* + 1) as follows:

(2) $$W_{\left(i,\;j+1\right)}=W_{\left(i,\;j\right)}-a\;\nabla C_{LOSS}\;\left(W_{\left(i,\;j\right)}\right)$$

where *i* and *j* represent the global and local iteration, respectively, $\nabla C_{LOSS}\;\left(W_{\left(i,\;j\right)}\right)$ is the gradient of $C_{LOSS}\;\left(W_{\left(i,\;j\right)}\right)$, and a represents the learning rate.

After the clients have completed the previously defined locally iterated *C_LOSS_* of the model training, they will communicate the final parameters to the central server so that they may be aggregated. The conclusion of a global iteration is signaled by the completion of global server aggregation, and at the beginning of the subsequent global iteration, the client will download the most recent version of the global parameter. When the set number of global iterations has been achieved, the final parameter W will have been gained.

Algorithm 1 is a pseudo-code representation of the DMFL_Net framework for training an FL-based COVID-19 identification model.
**Algorithm 1:** Proposed DMFL_Net**Parameters** Job = J, Decision Time = D_T_, Federated Learning steps = FL_STEPS_, Maximum Accuracy = A_MAX_, Training Time = T_TIME_, Waiting Time = W_TIME_, Model = M, Accuracy = A_ACC_, Model = M, Epochs = E, Learning rate = a, Batch Size = B_SIZE_
⋯/*Client-side process*/
1. **Set:** Clients (ServerURL)2. **J = download** (ServerURL)3. Decode (J) = (D_T_, M)4. **Starts:** FL_STEPS_ = 05. **Initialize:** a, B_SIZE_, E6. **While (**FL_STEPS_ < D_T_**):**7.⋯⋯**Train_Local_Model** (M, a, B_SIZE_, E) = T_TIME_, A_ACC_8.⋯⋯**Send** (T_TIME_, A_MAX_) = ServerURL9.⋯⋯**If** (A_ACC_ > A_MAX_):10.⋯⋯**Upload** (M) 11.⋯⋯**End**12. FL_STEPS_ ++13.·**End**14.·/*Server-side process*/15.·**Initialize:** W_TIME_, A_MAX_, M, D_T_16.·**While** (True):17.⋯·**Receive_Model_Update:** T_TIME_, Update (T_TIME_) = W_TIME_18.⋯·**Client_Local_Model =** Receive_Model_Update19.⋯⋯⋯**If (**W_TIME_ ends) == true:20.⋯⋯⋯Aggregate (Client_Local_Model**) =** M21.⋯⋯⋯A_MAX_ = Assess (M)22.⋯⋯⋯Dispatch (M)23.⋯⋯⋯**End**24. **End**

### 3.6. Performance Evaluation

In this work, we used three DNN models such as DenseNet-169, VGG-16, and VGG-19 for the classification of COVID-19 from four chest diseases. Thus, a confusion matrix was used to measure the performance of these DNN models in terms of many metrics, e.g., accuracy (ACC), precision (PRE), recall (REC), specificity (SPF), and F1-measure. The following Equations (3)–(7) are used to measure these metrics:

(3) $$ACC=\frac{TP+TN}{TP+TN+FP+FN}$$

(4) $$PRE=\frac{TP}{TP+FP}$$

(5) $$REC=\frac{TP}{TP+FN}$$

(6) $$SPF=\frac{TN}{TN+FP}$$

(7) $$F1-score=2\times \left(\frac{PRE\times REC}{PRE+REC}\right)$$

## 4. Results and Discussions

This study uses FL for the diagnosis of a variety of chest disorders, including COVID-19, Pneu, TB, PneuTh, and normal, with the aim to enhance communication efficiency [73,74,75] and model performance in terms of ACC, PRE, REC, SPF, and F1-measure. For the present study, 100 rounds were used in the experimentation process. The test was done on a Windows PC equipped with 32GB of RAM and a GPU from NVIDIA GeForce GTX 1070 11GB.

### 4.1. Experimental Configurations

As can be seen in Table 3, the trials involved one central server and three separate clients, each of which has its unique configurations.

We used the obtained data, which included 101,017 CXR images, and divided them as follows: 33,600 images were used for the training set, and 4200 images were used for the test set. As can be seen in Table 4, we establish different data sets of varying sizes for each client.

### 4.2. Comparison of DMFL_Net Model Accuracy with Default FL Configuration

Experiments were carried out using three distinct models, namely VGG-16, VGG-19, and DenseNet-169. To ensure that the DMFL_Net is operating appropriately, the models are trained with the six distinct varieties of data sets that are outlined in Table 4. Extensive experimentation has been performed and the outcomes attained by the DMFL_Net model are compared with the FL system’s default setup. Table 5, Table 6 and Table 7 present a detailed summary of the results obtained by the proposed DMFL_Net.

We distributed the dataset into different parts for each client separately. We have applied the DMFL_Net + VGG-16 model in 500 CXR images for the classification of COVID-19 from chest diseases. DMFL_Net + VGG-16 model achieved an ACC of 87.57%. Similarly, we also applied the default FL + VGG-16 model on the same dataset and achieved an ACC of 80.21%, which is less than the DMFL_Net + VGG-16 model. Furthermore, we applied CXR 1500 dataset for both DMFL_Net + VGG-16 model and the default FL + VGG-16 model, and DMFL_Net + VGG-16 showed significant results as compared to the default FL+ VGG-16. The detailed results are presented in Table 5.

Table 6 demonstrates that the DMFL_Net + VGG-19 model is used for 1000 CXR images to classify COVID-19 from chest illnesses. The DMFL_Net + VGG-19 model achieved 87.89% accuracy, 87.21% of PRE, and 87.25% of REC. Similar to DMFL_Net + VGG-19 model, we also applied the default FL + VGG-16 model to the same dataset and attained an ACC of 84.00%. Moreover, we applied the CXR 2000 dataset to both the DMFL_Net + VGG-19 model and the default FL + VGG-19 model, with DMFL_Net + VGG-19 producing significantly better results than the default.

**Figure 4:**
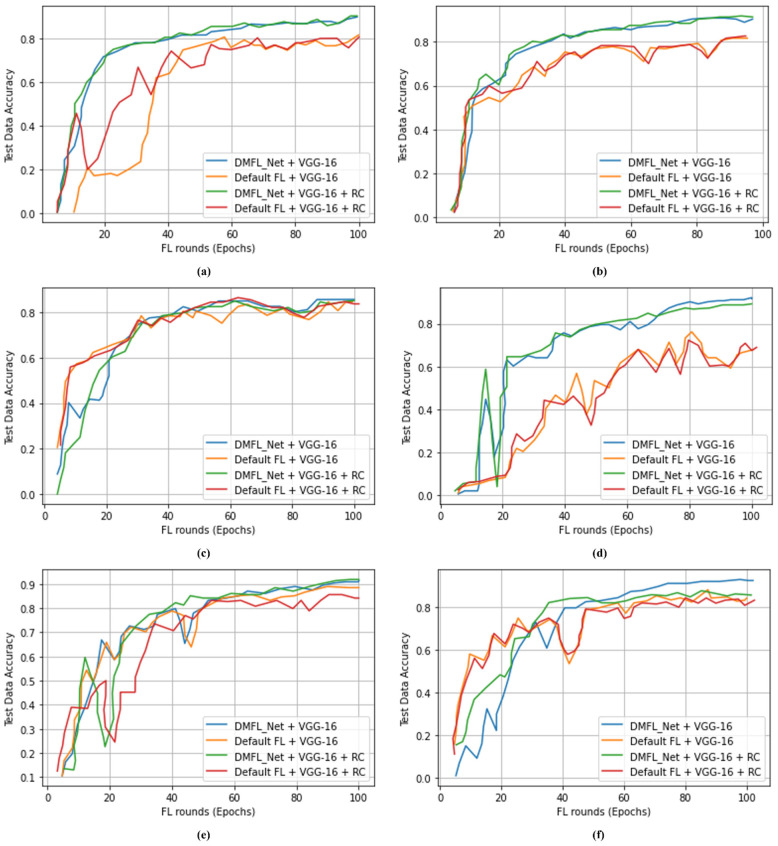
Accuracy of DMFL_Net with VGG-16; (**a**) 500 images; (**b**) 800 images; (**c**) 1200 images; (**d**) 1500 images; (**e**) 1800 images; (**f**) 2000 images.

**Figure 5:**
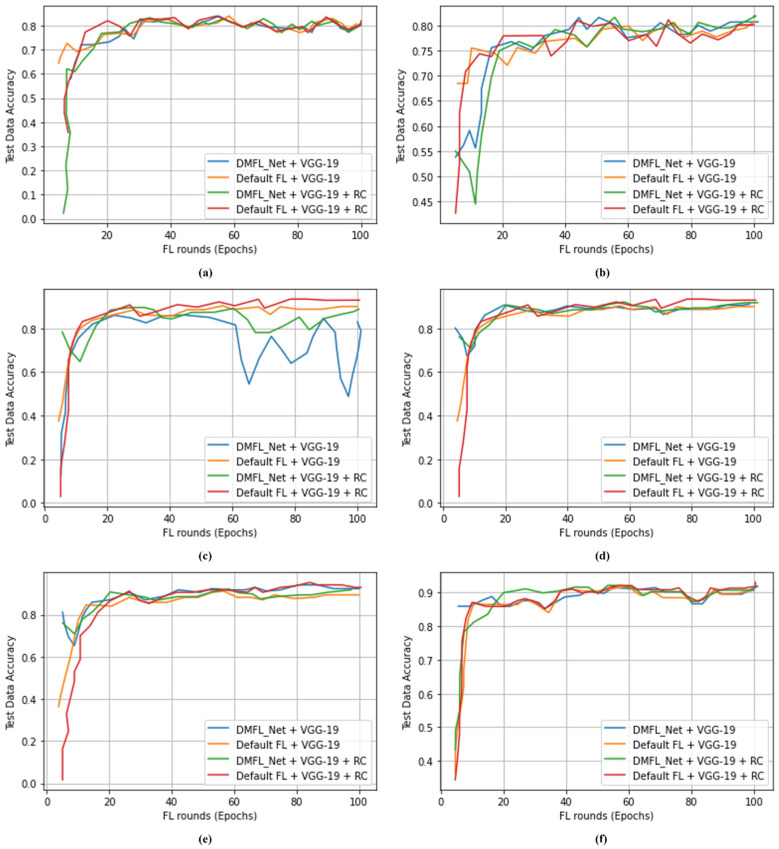
Accuracy of DMFL_Net with VGG-19; (**a**) 1000 images; (**b**) 1600 images; (**c**) 2400 images; (**d**) 3000 images; (**e**) 4000 images; (**f**) 5000 images.

**Figure 6:**
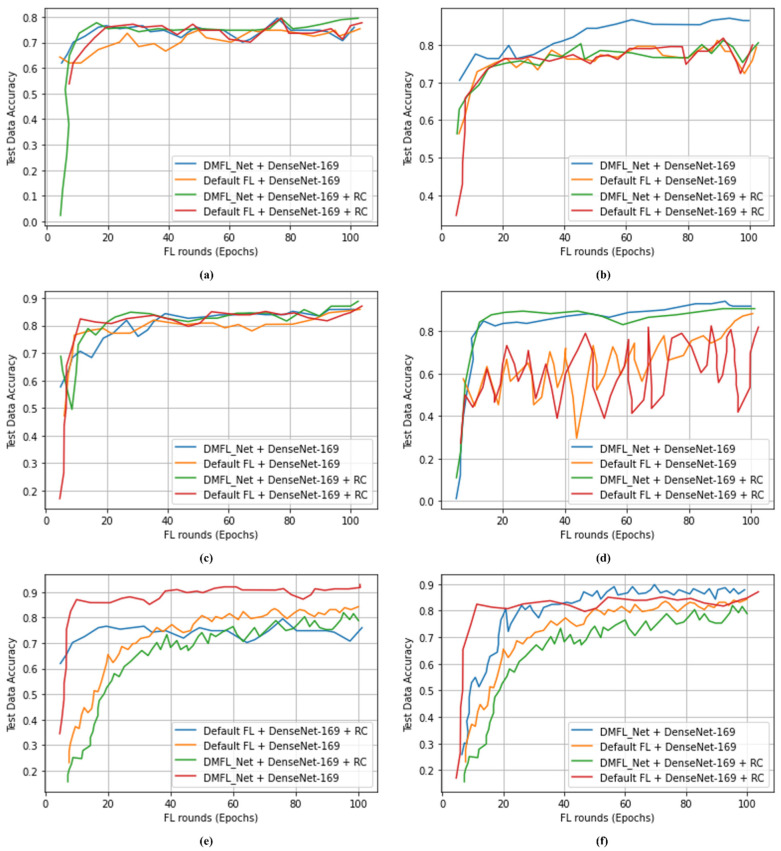
Accuracy of DMFL_Net with VGG-19; (**a**) 1000 images; (**b**) 1600 images; (**c**) 2400 images; (**d**) 3000 images; (**e**) 4000 images; (**f**) 5000 images.

Table 7 indicates that 1000 CXR images were classified using the DMFL_Net + DenseNet-169 model to differentiate COVID-19 from chest diseases. The proposed DMFL_Net + DenseNet-169 model obtained an accuracy of 98.45%, a PRE of 98.40%, a REC of 98.42, an SPF of 98.41%, and an F1-measure of 98.44%. The proposed DMFL_Net + DenseNet-169 produced superior results as compared to default FL approaches. Figure 4, Figure 5 and Figure 6 accordingly show the results that were obtained using the various types of models. The proposed DMFL_Net achieved a higher level of accuracy than the setting that is considered to be the standard for FL. In addition to this, interference was incorporated into the testing group of each model’s data set. Because of this interference, TL models that had a negative diagnosis ended up being incorrectly classified as having a positive result for COVID-19. The fact that the model trained by DMFL_Net may still generate relatively consistent results and higher accuracy in comparison to the default option illustrates that the proposed DMFL_Net can ensure robustness and communication (RC) [76]. In addition to this, we evaluated the precision of each model category by applying it to the randomly cropped test set. The findings are presented in Figure 4, Figure 5 and Figure 6.

According to the findings of testing groups, the suggested DMFL_Net achieves a higher level of accuracy than the FL option that is used by default. The DMFL_Net with DenseNet-169 achieves an accuracy of 98.45% which is relatively higher than the results produced by the default FL settings. The results reveal that the suggested DMFL_Net setup performs better than the default FL configuration on real-world data sets.

### 4.3. DMFL_Net Training Time

The length of the training sessions used in the trials was logged to evaluate how well the proposed DMFL_Net performed during its training phase. The maximum network speed for uploading and downloading models is 12 MB per second, and the number of training epochs for each client is set to 100.

**Figure 7:**
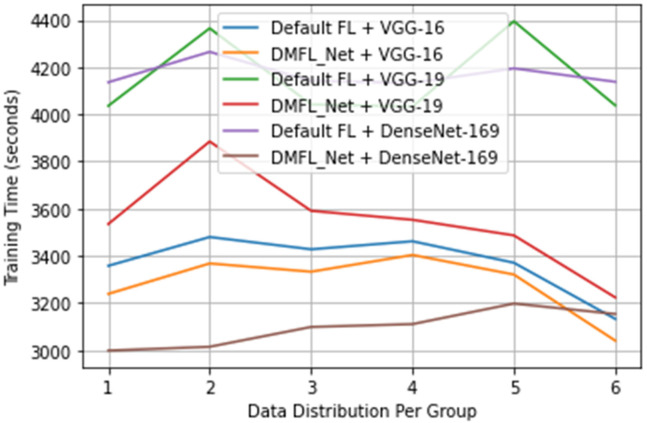
DMFL_Net training time.

Figure 7 presents the data gathered regarding the total amount of time spent on training. According to the findings, the suggested DMFL_Net did not cut down on the amount of time needed for training for VGG-16; however, it did for VGG-19 and DenseNet-169. The amount of time required for training VGG-19 was cut by 30–35 min, whilst the amount of time required for training DenseNet-169 was cut by 15–20 min. After finding in our previous experiments that the suggested DMFL_Net did not shorten the amount of time it took for the VGG-16 network to train, we are now investigating the factors that could have an impact on this. After determining the length of time necessary for the transmission of a single model, we concluded that the VGG-16 had a lower total number of parameters compared to the other two networks. Therefore, the VGG-16 needed less time for model transmission (an average of 3.7 s), which resulted in there being no change in the amount of time needed for training. On the other side, VGG-19 and DenseNet-169 have a greater number of parameters, which necessitated a longer period to communicate model updates. It would appear that there has been a major improvement in the efficiency of communication between these two networks as a result. If the network had been flawed and the model had a high number of parameters, we might have concluded that putting the DMFL_Net technique into practice could drastically cut down on the amount of time that is required for training.

### 4.4. DMFL_Net Communication Efficiency

**Figure 8:**
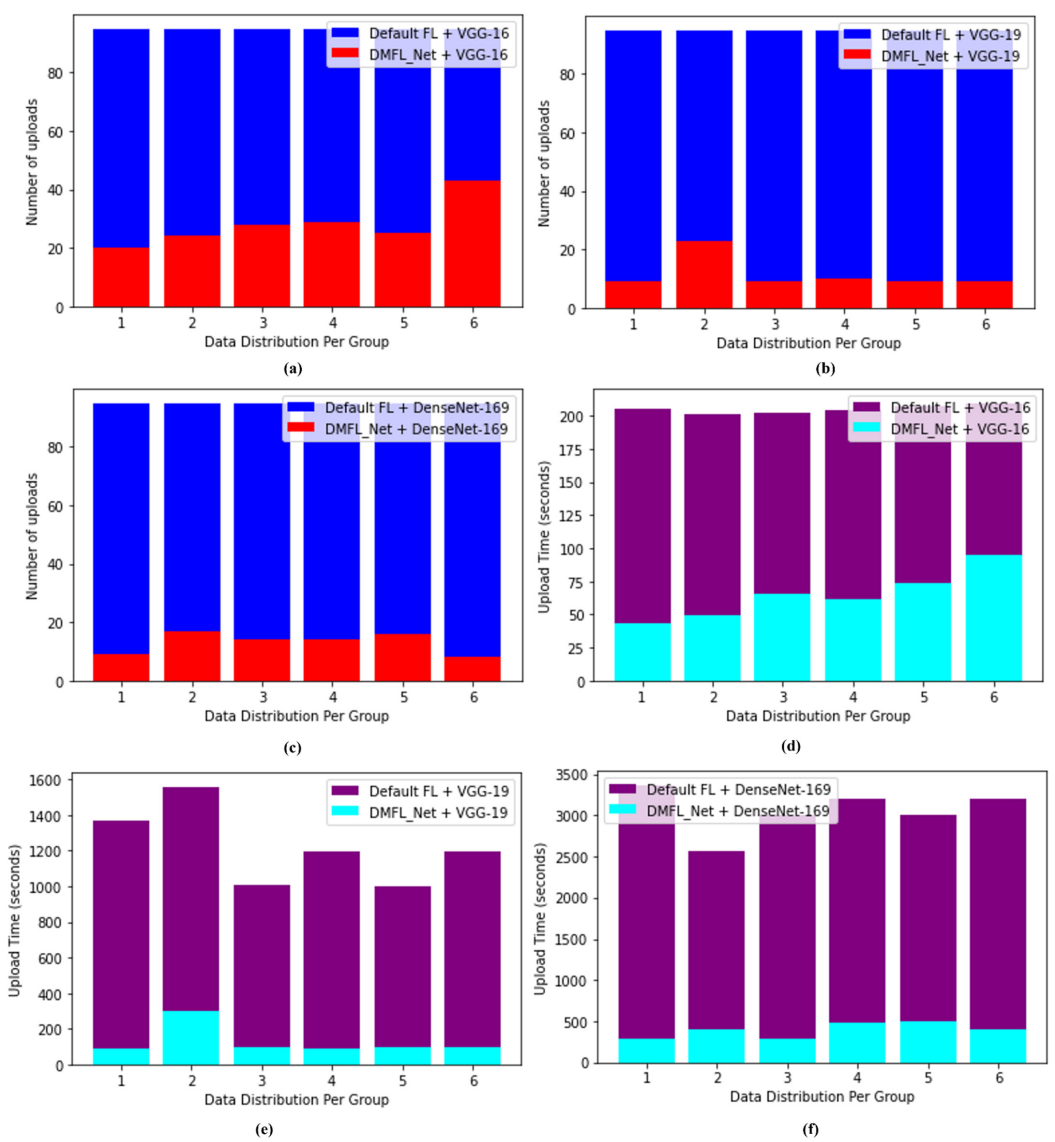
DMFL_Net upload number with (**a**) VGG-16; (**b**) VGG-19; and (**c**) DenseNet-169 and DMFL_Net upload time with (**d**) VGG-16; (**e**) VGG-19; and (**f**) DenseNet-169.

To evaluate the effect that DMFL_Net has had on communication, we counted the number of uploads and timed how long each one took, as shown in Figure 8. The total number of three clients was represented by both the quantity and duration of time spent gathering uploads in this location, which worked out to 15 times for each client and 40 times altogether. When compared to the default settings of FL for the VGG-16, the DMFL_Net model’s upload number was reduced by an average of 50, which resulted in a reduction of 120–180 s in the amount of time required for the upload (one-third of the default setting of FL time). The upload number of DMFL_Net decreased by an average of 70 for VGG-19, which translated to a decrease in upload time of between 1000 and 1300 s, which is equal to one-tenth of the duration of the default FL. The total number of uploads to DMFL_Net fell by 78 on average for DenseNet-169. Because of this, the drop-in upload time was between 4100 and 4300 s, which was equivalent to 1/16 of the FL time given by default. In light of the findings, we can conclude that DMFL_Net can lessen the burden of communication overhead by requiring less model uploading. The reduction is not noticeable in models such as the VGG-16, which has a straightforward structure and a limited number of parameters (approximately one-third of the default setting of FL). However, the effects of DMFL_Net are more pronounced when dealing with complex models that contain a greater number of parameters (VGG-19 and DenseNet-169). It cuts setting time to 1/10 and 1/16 of the standard FL value, respectively, which is a significant improvement.

### 4.5. Grad-Cam Visualization of DMFL_Net

**Figure 9:**
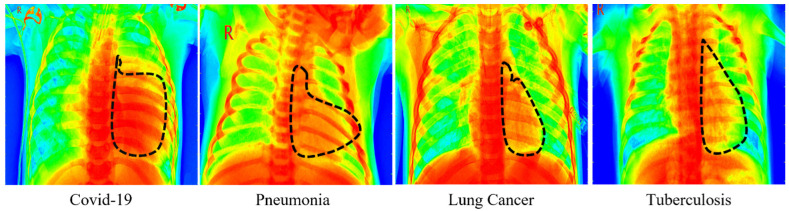
Grad-cam of four chest diseases.

We employed three TL models to perform a visual explanation of the DMFL_Net model, and the results are displayed in Figure 9 using Grad-Cam. The dotted line represents the infected portion of the chest recognized by the DMFL_Net model.

### 4.6. Comparison with State-of-the-Art

Numerous studies, such as [50,71,72,73,74,75], have been carried out to identify COVID-19; however, these methodologies do not make use of data sharing to construct an improved prediction model [77,78]. However, several of the algorithms used GAN in conjunction with data augmentation to generate fake CXR images. The reliability and the effectiveness of such methods cannot be guaranteed when it comes to medical CXR images. The examination of the data was difficult because there was only a small amount of patient information [69]. To develop a more accurate prediction model, the model that we have proposed gathers extensive amounts of data that were collected from publicly available sources [57,58,59,60,61,62,63,64,65,66]. To begin, we examined the state-of-the-art research in conjunction with the DL models outlined in Table 8. In addition, we examined how well FL performed in comparison to the most recent iterations of the DNN models, such as DenseNet-169, VGG-16, and VGG-19 [79,80]. The findings suggested that the accuracy was comparable regardless of whether the local model was trained with the entire dataset or by dividing the data among several hospitals and combining the model weights with the help of DMFL_Net.

### 4.7. Discussions

The classification of COVID-19 offered in this work makes use of CXR images as an automated supplement to the diagnostic procedure that is now considered the gold standard reverse transcription-polymerase chain reaction (RT-PCR) [81,82,83]. CXR images are substantially more readily available than RT-PCR, can determine a patient’s prognosis at any stage in the evolution of a disease, provide test results in a short amount of time, and significantly enhance the amount of daily testing capacity [80,81,82,83,84]. The DNN [82] model originated from an FL training session utilizing an aggregated version of the model. Our DMFL_Net + DenseNet-169 algorithm can be used as a completely autonomous method for differential diagnosis between normal lung aspect, COVID-19, LC, TB, PneuTh, and Pneu. This differential diagnosis is based solely on the aspect of the CXR image that is being analyzed. In addition, because the three clients who took part in the training process did not reveal any samples from the dataset, our method can be used as a collaborative [79] and decision-making methodology between medical institutions to construct a shared DNN model for COVID-19 identification without disclosing confidential data. This paper presents a DMFL_Net approach for classifying COVID-19 from various chest ailments using DNN models. The training process can be carried out over multiple machines, each of which possesses its CXR images dataset. A centralized server acts as the coordinator for all of the machines to produce a global model [76]. When the model is obtained, the weights of the model are transmitted to each machine so that the locally stored model can be brought up to date [84]. Each of the FL entities that were deployed locally in DMFL_Net was prepared with three different DNN models, specifically VGG-16, VGG-19, and DenseNet-169. The experimental design used in this study is outlined in Table 3. The fully connected layers (FCL) of DenseNet-169, VGG-16, and 19 were eliminated and then replaced with an FCL that consisted of 512 neurons and that used the rectified linear unit (ReLU) as its activation function, as well as an FCL that consisted of six neurons and used Softmax as its activation function. To ensure that the DL model’s convolutional layers remained static throughout the training process, they were frozen. The outputs of the aggregated model’s six neurons, all of which were multiclass models, were: Normal, COVID-19, LC, TB, and PneuTh. The categories ACC, PRE, SPF, REC, and F1-measure were observed to conduct an accurate evaluation of the performance of the aggregated model. The proposed DMFL_Net + DenseNet-169 was also compared with the default FL settings. The DMFL Net + DenseNet-169 model that was proposed obtained an accuracy of 98.45% and surpassed other approaches in the classification of COVID-19 from four chest disorders. This was accomplished while successfully maintaining the privacy of the data among a variety of clients.

This study had a limitation in that previous studies investigated the temporal changes (i.e., variation in COVID-19 causes a shift in patient demographics or radiographic characteristics) that resulted in COVID-19 diagnostic model bias and performance drift; however, the purpose of this study was to investigate data heterogeneity and model federation.

## 5. Conclusions

Traditional AI systems that are now in use often need to use centralized data storage and training to construct predictive models, which both increase the complexity of computing and threaten users’ privacy. This study offers a potential solution to the problem in the form of the DMFL_Net design, which aims to enhance the perception of multiple CXR image sources. It protects both the confidentiality and the security of the data when they are transferred between hospitals. The DNN models (DenseNet-169, VGG-16, and VGG-19) are used for effective feature extraction and classification to locate COVID-19 among the many different public CXR datasets. In addition, the use of FL for collaborative training in hospitals, which was supported by unpredictable encryption keys throughout the process of data retrieval and sharing, has improved trust in the maintenance of privacy and security. An exhaustive testing and comparing process using some different DNN algorithms has been carried out. The performance of DMFL_Net was evaluated and contrasted with that of the standard FL configuration. According to the results, DMFL_Net showed the best performance in terms of ACC (98.45%), PRE (98.40%), REC (98.42%), and F1-measure (98.44%). Furthermore, this method was more significant than the others in terms of trustworthiness and datasets. In the future, we will be in a position to provide a single network through which healthcare practitioners will be able to freely exchange patient records and train vision transformers to examine different privacy strategies.

## Figures and Tables

**Table 1:** Insightful reviews of recent literature on FL and cutting-edge algorithm design methods.

Ref	Disease Classification	Models	Contribution	Data Privacy and Distribution	Limitation	Outcomes
[2]	COVID-19 and Pneumonia	Clustered FL	It presents a framework for collaborative learning for intelligent edge device visual data processing by developing a multi-modal ML system that can identify COVID-19 in X-ray and Ultrasound images.	Yes	The number of samples per customer changes, which is a crucial factor in clustered FL performance.	F1-score = 76.0% and Recall = 82%
[3]	COVID-19	FedMA, deep CNN and LSTM	FedMA develops the global shared model by building it up one layer at a time, using the feature extraction signatures of undetected parts.	Yes	Protection of data confidentiality	Accuracy = 94.70%
[49]	COVID-19	CNN	A shared FL framework for filtering COVID-19 from CXR images with DL, without sharing patient data.	Yes	The problem of inconsistent data must be solved.	Accuracy = 97%
[51]	COVID-19 and Pneumonia	DenseNet-121 + FL	The suggested approach enables users to identify COVID-19 in a matter of seconds from the upload of a single chest X-ray picture.	Yes	The problem of inconsistent data must be solved.	Accuracy = 97.51%
[53]	COVID-19 and Viral Pneumonia	CNN	The current gold standard for screening for COVID-19 patients is RT-PCR testing, which is a highly time-consuming, labor-intensive, and complex manual approach. Thus, DCNN trained on chest X-rays can ascertain this phenomenon.	No	Conventional training algorithms have the drawbacks of slow convergence speed and local optima stagnation.	Accuracy = 90.64%
[54]	COVID-19	pretrained DenseNet-201	An optimal DL method as a way of separating individuals with COVID-19 infection from healthy people	No	The problem of inconsistent data must be solved.	Accuracy = 94.76%
[37]	COVID-19	CNN	Conceived of a distributed system whereby users may pool their gradients to boost the safety of shared data.	Yes	Underfitting occurs when a network’s size is inadequate.	-

**Table 2:** Summary of the datasets used in this study.

Labels	COVID-19	LC	TB	PneuTh	Pneu	Normal	Total
CXR	3301	5000	700	2793	3867	1349	17,017
CXR augmentation	7000	7000	7000	7000	7000	7000	42,000
Training (80%)	5600	5600	5600	5600	5600	5600	33,600
Validation (10%)	700	700	700	700	700	700	4200
Testing (10%)	700	700	700	700	700	700	4200
Total	17,301	19,000	14,700	16,793	17,867	15,349	101,017

**Table 3:** Experimental configuration.

Modes	GPU	RAM	Python	CUDA
Client 1	GTX 1070	11 GB	3.9	11.8
Client 2	GTX 1070	11 GB	3.9	11.8
Client 3	GTX 1070	11 GB	3.9	11.8
Server	GTX 1080	11 GB	3.9	10.1

**Table 4:** Division of the CXR images into six groups. Each group contains different CXR images and assigned these images to three different clients.

Types	Client 1	Size (MB)	Client 2	Size (MB)	Client 3	Size (MB)	Total Images	Total Size (MB)
1	500	70.81	1000	100.2	2000	165	3500	336.01
2	800	90.25	1600	136.93	3000	190.96	5400	418.14
3	1200	121.35	2400	170.85	3200	195.98	6800	488.18
4	1500	135.69	3000	190.96	3500	205.95	8000	532.6
5	1000	150	4000	205.95	3800	235.96	8800	591.91
6	2000	165	4200	225.50	4200	305.56	10,400	470.56

**Table 5:** Results were obtained by using DMFL_Net and default FL settings with VGG-16.

Models	CXR Images	Accuracy	Precision	Recall	Specificity	F1-Measure
DMFL_Net + VGG-16	500	87.57%	86.71%	86.23%	86.86%	86.99%
Default FL + VGG-16		80.21%	79.92%	79.79%	80.01%	80.09%
DMFL_Net + VGG-16	800	87.30%	86.13%	86.14%	86.29%	87.23%
Default FL + VGG-16		81.26%	80.96%	80.94%	80.96%	81.07%
DMFL_Net + VGG-16	1200	85.01%	84.92%	84.97%	84.92%	85.00%
Default FL + VGG-16		84.91%	84.82%	84.79%	84.87%	84.89%
DMFL_Net + VGG-16	1500	88.12%	88.02%	88.05%	88.09%	88.09%
Default FL + VGG-16		64.79%	64.24%	64.41%	64.47%	64.57%
DMFL_Net + VGG-16	1000	91.01%	90.99%	90.97%	90.96%	91.00%
Default FL + VGG-16		89.29%	89.03%	89.07%	89.19%	89.15%
DMFL_Net + VGG-16	2000	88.33%	88.12%	88.14%	88.25%	88.22%
Default FL + VGG-16		82.19%	82.01%	81.98%	82.09%	82.07%

**Table 6:** Results were obtained by using DMFL_Net and default FL settings with VGG-19.

Models	CXR Images	Accuracy	Precision	Recall	Specificity	F1-Measure
DMFL_Net + VGG-19	500	80.96%	80.06%	80.19%	80.39%	80.47%
Default FL + VGG-19		80.01%	79.99%	80.00%	80.00%	80.01%
DMFL_Net + VGG-19	800	81.95%	81.15%	81.37%	81.42%	81.79%
Default FL + VGG-19		79.18%	79.45%	79.55%	79.78%	79.95%
DMFL_Net + VGG-19	1200	90.15%	90.07%	90.09%	90.12%	90.14%
Default FL + VGG-19		85.26%	85.01%	85.13%	85.17%	85.19%
DMFL_Net + VGG-19	1500	87.78%	87.38%	87.59%	87.67%	87.71%
Default FL + VGG-19		85.23%	85.03%	85.13%	85.12%	85.19%
DMFL_Net + VGG-19	1000	87.89%	87.21%	87.35%	87.49%	87.59%
Default FL + VGG-19		84.00%	83.98%	83.99%	84.00%	84.00%
DMFL_Net + VGG-19	2000	92.25%	92.05%	92.11%	92.16%	92.21%
Default FL + VGG-19		90.03%	90.01%	90.00%	90.02%	90.03%

**Table 7:** Results were obtained by using DMFL_Net and default FL settings with DenseNet-169.

Models	CXR Images	Accuracy	Precision	Recall	Specificity	F1-Measure
DMFL_Net + DenseNet-169	500	78.89%	78.49%	78.33%	78.42%	78.75%
Default FL + DenseNet-169		75.25%	75.12%	75.19%	75.21%	75.22%
DMFL_Net + DenseNet-169	800	88.48%	88.21%	88.19%	88.29%	88.37%
Default FL + DenseNet-169		80.89%	80.29%	80.34%	80.43%	80.69%
DMFL_Net + DenseNet-169	1200	92.69%	92.32%	92.27%	92.31%	92.47%
Default FL + DenseNet-169		89.26%	89.06%	89.13%	89.15%	89.19%
DMFL_Net + DenseNet-169	1500	94.87%	94.80%	94.81%	94.84%	94.83%
Default FL + DenseNet-169		87.78%	87.58%	87.63%	87.69%	87.72%
DMFL_Net + DenseNet-169	1000	98.45%	98.40%	98.42%	98.41%	98.44%
Default FL + DenseNet-169		90.40%	90.36%	90.34%	90.37%	90.38%
DMFL_Net + DenseNet-169	2000	97.56%	97.42%	97.44%	97.41%	97.50%
Default FL + DenseNet-169		90.45%	90.40%	90.39%	90.41%	90.42%

**Table 8:** Comparison of the proposed model with recent studies.

Ref	Design Methods	Disease Classification	Accuracy	Data Sharing	Privacy
[71]	2D UNET++	COVID-19 and Pneumonia	96.21%	No	No
[76]	Attention module + ResNet-50	COVID-19 and Pneumonia	93.30%	No	No
[77]	FL + CapsNet	COVID-19	89.00%	Yes	Yes
[78]	CNN	COVID-19	85.40%	No	No
[1]	Genetic CFL	COVID-19	91.01%	Yes	Yes
[2]	Clustered FL	COVID-19	95.15%	Yes	Yes
Ours	DMFL_Net + DenseNet	COVID-19, LC, TB, PneuTh, Pneu.	98.45%	Yes	Yes

## Data Availability

Not applicable.
